# Increased proinflammatory responses from asthmatic human airway smooth muscle cells in response to rhinovirus infection

**DOI:** 10.1186/1465-9921-7-71

**Published:** 2006-05-03

**Authors:** Brian GG Oliver, Sebastian L Johnston, Melissa Baraket, Janette K Burgess, Nicholas JC King, Michael Roth, Sam Lim, Judith L Black

**Affiliations:** 1 Department of Pharmacology, University of Sydney, NSW, 2006, Australia; 2 Department of Respiratory Medicine, National Heart and Lung Institute, Imperial College London, UK; 3 Woolcock Institute for Medical Research, NSW 2006, Australia; 4 Department of Pathology, University of Sydney, NSW, 2006, Australia; 5 Pulmonary Cell Research, Dept. Research, University Hospital Basel, CH-4031 Basel, Switzerland; 6 ANZAC Research Institute, University of Sydney, Sydney, Australia

## Abstract

**Background:**

Exacerbations of asthma are associated with viral respiratory tract infections, of which rhinoviruses (RV) are the predominant virus type. Airway smooth muscle is important in asthma pathogenesis, however little is known about the potential interaction of RV and human airway smooth muscle cells (HASM). We hypothesised that rhinovirus induction of inflammatory cytokine release from airway smooth muscle is augmented and differentially regulated in asthmatic compared to normal HASM cells.

**Methods:**

HASM cells, isolated from either asthmatic or non-asthmatic subjects, were infected with rhinovirus. Cytokine production was assayed by ELISA, ICAM-1 cell surface expression was assessed by FACS, and the transcription regulation of IL-6 was measured by luciferase activity.

**Results:**

RV-induced IL-6 release was significantly greater in HASM cells derived from asthmatic subjects compared to non-asthmatic subjects. This response was RV specific, as 5% serum- induced IL-6 release was not different in the two cell types. Whilst serum stimulated IL-8 production in cells from both subject groups, RV induced IL-8 production in only asthmatic derived HASM cells. The transcriptional induction of IL-6 was differentially regulated via C/EBP in the asthmatic and NF-κB + AP-1 in the non-asthmatic HASM cells.

**Conclusion:**

This study demonstrates augmentation and differential transcriptional regulation of RV specific innate immune response in HASM cells derived from asthmatic and non-asthmatics, and may give valuable insight into the mechanisms of RV-induced asthma exacerbations.

## Background

Asthma exacerbation is the major contributor to morbidity, mortality and health care costs associated with this highly prevalent disease. Approximately 80% of asthma exacerbations in children [1-4] and about 70% in adults [5,6] are associated with respiratory viral infections. Rhinovirus (RV) is by far the most common virus type associated with asthma exacerbations [3,7,8]. RV can infect the lower respiratory tract, as demonstrated by Papadopoulos and co-workers (2000), who used in-situ hybridization to detect RV infection of both bronchial epithelial and underlying submucosal cells in biopsies obtained from the lower airways [9]. Although the authors did not identify the infected submucosal cells it is likely that they would have been mesenchymal in origin, eg fibroblasts and/or smooth muscle cells, as identified by the histological representation of positive signal for infection demonstrated in this paper.

Normally the bronchial epithelium forms a barrier between the airway lumen and the underlying cells. However, epithelial cells from asthmatic subjects have impaired RV-induced apoptosis and increased RV replication and cell necrosis in comparison to cells derived from non-asthmatic subjects [10]. Furthermore, in the asthmatic airway there may be desquamation of the epithelial cell layer [11], and increased smooth muscle mass [12]. These asthma specific structural changes, in combination with RV-induced necrosis of bronchial epithelial cells, increase the likelihood of RV infecting the underlying smooth muscle during a naturally acquired RV infection in asthmatic subjects.

Human airway smooth muscle (HASM) cells are actively involved in maintaining the local immune environment, through the production of a wide variety of immunomodulatory factors [13], and modulation of their cell surface receptors [14-16]. The host-mediated immune response to RV is important in viral clearance from the lower respiratory tract. Following RV infection, lower airway neutrophilia occurs [17], which is likely to be as a result of RV-induced chemokine release. IL-8 is a potent chemotactic agent for neutrophils [18], in addition to activating several cell types found in the lungs. IL-6 is a complex and pleiotropic cytokine which has many functions and may contribute to the progression of asthma, since it polarizes T-helper cells towards a T-helper 2 phenotype [19]. Furthermore, IL-6 induces differentiation of T cells, B cells and macrophages, in addition to contributing to the recruitment of mononuclear cells and neutrophils [20,21]. Previous experiments, mainly carried out using rabbit airway smooth muscle cells and two HASM cell lines derived from non-asthmatic healthy lung donors, have shown that RV infection induces the production of interleukin (IL)-1β and IL-5 [22,23].

Since it has been previously suggested that RV infection of HASM cells mediates cytokine production [22], and since major differences in innate responses of bronchial epithelial cells from asthmatic and normal subjects to RV infection have been recently demonstrated [10], we hypothesised that RV induction of inflammatory cytokine release is augmented in primary HASM cells from asthmatic compared with normal subjects. Having found this to be the case, and with the knowledge that a transcription factor which can bind to the IL-6 promoter region, C/EBP-α, is absent from asthmatic but not normal HASM cells [24] we then investigated transcriptional regulation of IL-6 to determine whether its expression is differentially regulated in asthmatic compared to normal HASM cells.

## Methods

### Patient demographics

HASM cells were isolated from airway muscle bundles obtained from 22 asthmatic subjects [mean age 23 years, range 18–33]), and 29 non-asthmatic subjects [mean age 55 years, range 16–74] of which 17 were undergoing lung resection for a tumour, 7 were undergoing transplantation (cystic fibrosis [n = 1], pulmonary fibrosis [n = 2], emphysema [n = 2], Eisenmenger's syndrome [n = 1], and Tetralogy of Fallot [n = 1]) and 5 were healthy. Four of the non-asthmatic subjects were females, and 23 were male, the age and sex of two of the non-asthmatic subjects were not available. Four of the asthmatic subjects were females, and 18 were males.

### RV propagation and titration

Stocks of human RV-16 were amplified by growth in Ohio HeLa cells as previously described [25]. In some experiments RV was UV-inactivated in 24 well plates containing 200 μl of viral stock per well, at a distance of 5 cm from a 30 W UV light source (germicidal lamp G30T8, Sankyo Denki, Japan), for 15 minutes. UV inactivation (UVi) of RV was shown to be effective by RV titration assay. HASM were infected with RV at a multiplicity of infection (MOI) of 4, 0.4 and 0.04. Following exposure to RV, virus release was assessed by titration assay [25]. Breifely, Rhinovirus levels were estimated by titrating serially log diluted concentrations of the cell free supernatant in quadruplicate upon Ohio HeLa cells. Ohio HeLa cells were seeded at a concentration of 1 × 10^5^cells/ml in 96 well plates. To this 50 μL of viral suspension or control medium were added. The plate was shaken for ten minutes at room temperature, and cultured for 4–6 days. After 3 days of culture, cells were assessed for cytopathic effect (CPE). The cells were then assessed for CPE every 24 hours, to ensure that cell death of control wells was not occurring and therefore biasing viral induced CPE. Viral concentration was determined as the lowest viral concentration which caused cytopathic effect in 50% of the wells (tissue culture infective dose 50 [TCID _50_]).

### Isolation and culture of human airway smooth muscle cells

HASM cells were isolated from bronchial tissue obtained from either bronchoscopy, lung tissue obtained from lung transplants, or lung tissue resected at thoracotomy, by microdissection. Ethical approval for the use of the lung tissue for in vitro experimentation was granted by the Human Ethics Committee of the University of Sydney, and the Central Sydney Area Health Service, and informed consent was received from all subjects. This isolation and culture of HASM cells was carried out according to a method described by Johnson et al 1995 [26]. HASM cells were used between passage 4–7. HASM cells were identified by morphology, and positive immunofluorescent staining with a specific α-smooth muscle actin antibody [26] and a calponin antibody [27]. HASM cells were seeded in 12 well plates at a density of 3.2 × 10^4 ^cells / ml in 5% FBS in DMEM (without the addition of antibiotics). For experiments using subconfluent cells, experimentation was carried out using HASM cells 24 hours post seeding, and confluent cells were obtained following 7 days of growth.

### RV infection

The medium bathing the HASM cells was removed and replaced with medium containing RV. The cells were incubated at 37°C with shaking every fifteen minutes, for one hour. The medium was removed and the cells were washed 3 times in sterile PBS, and 1 ml of DMEM (either 0.1% FBS or 5% FBS) added. Cells exposed to UV irradiated RV and cells with no virus exposure underwent the same infection procedure (with the absence of RV) as infected cells. Medium was harvested at 24 hours post infection, following centrifugation to remove non-adherent HASM cells, and stored at -80°C, for analysis by ELISA and RV titration assays. HASM cell viability was determined using manual cell counting and trypan blue exclusion assays.

### ELISA

ELISAs for eotaxin, tumour necrosis factor (TNF)-α, IL-6, IL-8, and interferon (IFN)-γ were purchased from R&D Systems Europe, Abingdon, UK. ELISAs were carried out according to the manufacturer's instructions. The detection limits of these assays were: 15.6 pg/ml for all except eotaxin (25 pg/ml).

### Flow cytometry

Flow cytometric analysis was performed to assess the cell surface expression of ICAM-1 (BD, North Ryde, Australia) in comparison to an isotype control antibody (BD).

### IL-6 promoter constructs – amplification and isolation

A plasmid containing a 651 bp fragment of the human IL-6 gene was kindly provided by Shigeru Katamine (Nagasaki University, Nagasaki, Japan). The IL-6 promoter constructs were designed as previously reported by Eickelberg et al (1999) [28]. Briefly, site-directed mutagenesis was used to inactivate various transcription factor binding sites. The AP-1 consensus sequence (positions 283 to 276, 5'-TGAGTCAC-3') was changed to 5'-TGCAGCAC-3'; the C/EBP consensus sequence (positions 154 to 146, 5'-TTGCACAAT-3') was changed to 5'-CCGTTCAAT-3'; and the NF-κB consensus sequence (positions 72 to 63, 5'-GGGATTTTCC-3') was changed to 5'-CTCATTTTCC-3'.

### Transient transfection of HASM cells

A commercially available kit (Effectene^® ^transfection reagent, Qiagen, Australia) was used. The use of a second plasmid to control for transfection efficiency is used by some researchers, however preliminary experiments indicated that transfection of HASM cells with the control Renilla luciferase reporter vector (Promega, Australia) in addition to the IL-6 promoter construct plasmids resulted in interference between the two plasmids, and therefore this practice was not continued. This effect has been demonstrated in other reports [29,30], and it is recommended that two plasmids should not be used in some systems. The manufacturer's transfection protocol was followed, with a ratio of DNA (0.5 μg/well) to Effectene^® ^transfection reagent of 1:4. Following incubation with the transfection mixture for 24 hours (in 5%FBS + DMEM), cells were washed twice with PBS, and 1 ml of 0.1% FBS in DMEM was added to each well, and then exposed to UVi RV (initial MOI of 4), or platelet derived growth factor (PDGF 10 μg/well), or infected with RV at MOI of 0.04. Control cells were cultured in the presence of 0.1% FBS in DMEM alone. Stimulation or infection was maintained for 24 hours, at which time cells and supernatant were harvested for determination of luciferase activity.

### Measurement of luciferase activity

Luciferase activity was determined using a commercially available kit (Dual-Luciferase^® ^reporter assay system, Promega, Australia) according to the manufacturer's instructions, using a Turner Biosystems luminometer, (Promega, Australia)

### Statistical methods and analysis of results

Data are presented as mean ± SEM. Data were subjected to two tailed Student's paired T test or repeated measures ANOVA with Dunnett's multiple comparison post test. Data derived from different donor populations were subjected to unpaired two-tailed Student's t-test. Data were analysed using GraphPad Prism version 4.00 for Windows (GraphPad Software, San Diego, California, USA). A probability level of 95% (p ≤ 0.05) was considered to be the threshold for statistical significance.

## Results

### Infection of HASM cells with RV induces cell death

**Figure 1:**
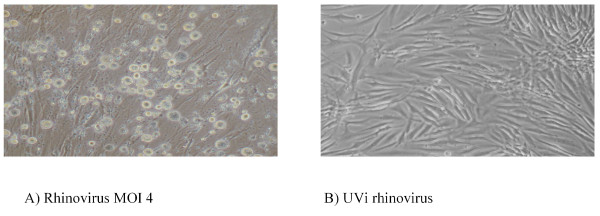
**Rhinovirus infection of HASM cells induces necrotic cell death**. Photomicrographs of HASM cells 24 hours post: A) Infection with rhinovirus at an MOI of 4, and B) exposure to UVi rhinovirus. The photomicrographs are of HASM cell derived from a single asthmatic patient, and are representative of the response observed in HASM cells derived from all asthmatic and non-asthmatic donors tested.

Firstly, we wished to confirm that RV is able to productively infect HASM cells. We compared the RV-induced rate of cell death in asthmatic and non-asthmatic HASM cells. Infection with RV-16 (MOI of 4) induced significant cell death in HASM cells derived from both asthmatic and non-asthmatic subjects, the viability of asthmatic cells was reduced to 45 ± 13% and non-asthmatic cells to 52 ± 11% by 24 hours post infection (p < 0.05 RV versus control, n = 3 for both). No difference in the RV-induced cell death between the cells derived from the two subject groups was observed (Figure 1A). There was no significant cell death in comparison to non-infected control cells with infection at lower virus concentrations (0.4 and 0.04 MOI). In contrast, no cell death was observed following exposure to UVi RV-16. (Figure 1B).

Infection was verified by titration assay of the tissue culture medium, with maximum RV-16 release occurring at 24 hours, decreasing at both 48 and 72 hours. When corrected for cell number, greater RV-16 production occurred in proliferating HASM cells compared to confluent cells. However, no difference in RV production was observed, with initial infection at a MOI of 4, between cells derived from asthmatics and non-asthmatics in both proliferating cells (asthmatic 46350 ± 15070 TCID_50_/ml and non-asthmatics 44220 ± 8820 TCID_50_/ml, p > 0.05 n = 6) and confluent cells (asthmatic 30010 ± 9187 TCID_50_/ml and non-asthmatics 37860 ± 19200 TCID_50_/ml, p > 0.05 n = 5). Infection was also verified using RT-PCR (data not shown).

### Distinct pro-inflammatory cytokine response to RV-16 infection in HASM cells derived from asthmatic and non-asthmatic subjects

To investigate whether RV infection of HASM cells induced augmented pro-inflammatory cytokine responses in asthmatic compared with non-asthmatic subjects, we next investigated IL-6, IL-8, eotaxin, TNF-α and IFN-γ secretion into the tissue culture medium in response to RV infection.

**Figure 2:**
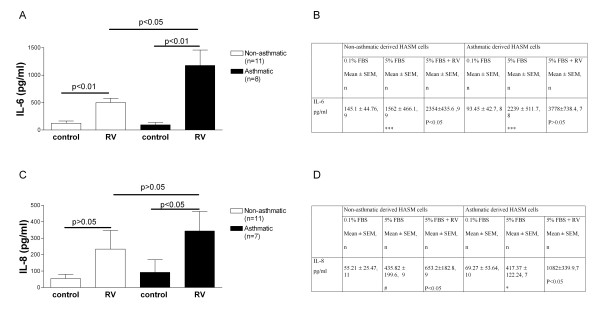
**Cytokine release from infected HASM cells**. A and C cytokine release in response to rhinovirus infection of HASM cells derived from non-asthmatic (open bars) and asthmatic (black bars) subjects, measured in the cell free tissue culture supernatant 24 hours post infection. B and D Stimulation of HASM cells with 5% FBS and concurrent RV infection. No significant difference was observed between IL-6 and IL-8 output in response to FBS stimulation between HASM cells derived from asthmatic and non-asthmatic volunteers. IL-6 and IL-8 were induced in both cell types in response to stimulation with 5% FBS, and concurrent infection with RV in the presence of 5% FBS induced IL-6 and IL-8 release in the non-asthmatic cells, and IL-8 in the non-asthmatic cells. # p = 0.05 0.1% FBS versus 5%FBS, * p < 0.05 0.1% FBS versus 5%FBS, *** p < 0.001 0.1% FBS versus 5%FBS, the P value indicates the level of significance between 5%FBS and 5%FBS + RV.

As shown in Figure 2A, infection with RV-16 (MOI of 4) significantly increased IL-6 secretion in both non-asthmatic (p < 0.001, n = 11) and asthmatic (p < 0.01, n = 8) -derived HASM cells in comparison to non-infected control cells 24 hours post infection. Furthermore, the RV induced release of IL-6 was 2.5 fold and significantly greater from cells obtained from asthmatic than non-asthmatic patients (asthmatic 1177 ± 281.5 n = 8 and non-asthmatic 501.0 ± 78.7 n = 11 p = 0.017). 5% FBS stimulated secretion of IL-6 in both asthmatic and non-asthmatic cells, and in contrast to RV-induced IL-6, no differences were found between the two subject groups (Figure 2B). Infection with RV-16 (MOI 4) and concurrent stimulation with 5% FBS significantly induced the release of IL-6 in comparison to 5% FBS alone in HASM cells derived from only non-asthmatic donors, whilst a non-significant trend towards increased production occurred in the asthmatic-derived cells (Figure 2B).

**Figure 3:**
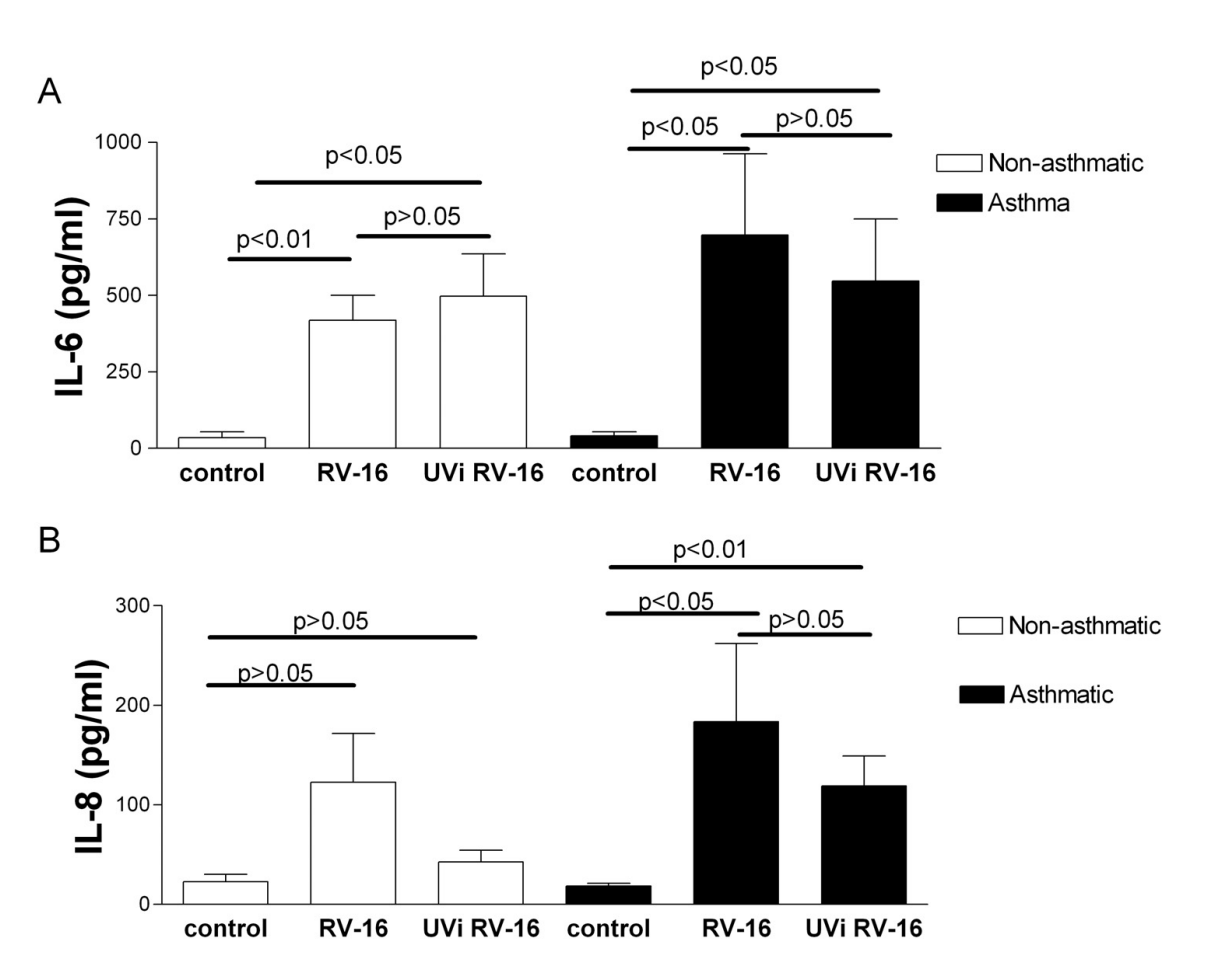
**UVi rhinovirus induces cytokine release from infected HASM cells**. Cytokine release in response to rhinovirus infection, and exposure to UVi rhinovirus (UVi), of HASM cells derived from non-asthmatic (open bars) and asthmatic (black bars) donors, measured in the cell free tissue culture supernatant 24 hours post infection. a) IL-6 release n = 8 non-asthmatic and n = 6 asthmatic derived HASM cells, b) IL-8 release n = 8 non-asthmatic and n = 9 asthmatic derived HASM cells For comparison infectious rhinovirus is shown upon each graph where the data originate from HASM cells derived from the same donor. Repeated measures ANOVA with Dunnett's Multiple Comparison Test post test.

As shown in Figure 2C, in non-asthmatic HASM cells RV infection caused an increase of IL-8 secretion, however, due to variation this difference did not become significant (n = 11). In contrast, RV-16 significantly increased the secretion of IL-8 significantly above non-infected asthmatic-derived HASM cells 24 hours post infection (p < 0.05, n = 8, Figure 3A). In contrast, stimulation with 5% FBS, a commonly used HASM cell stimulus [24,31], increased the production of IL-8 in both asthmatic and non-asthmatic derived HASM cells in comparison to constitutive release, and infection with RV-16 (MOI 4) and concurrent stimulation with 5% FBS significantly induced the release of IL-8 in comparison to 5% FBS alone in HASM cells derived from both asthmatic and non-asthmatic subjects. (Figure 2D).

The production of eotaxin was not induced following infection with RV-16 at an MOI of 4, when measured 24 hours post infection. Tumour necrosis factor α and IFN-γ were either not produced or below the limit of detection in HASM cells derived from both asthmatic and non-asthmatic subjects.

### UVi RV-16-induced secretion of IL-6 and IL-8

In a separate series of experiments we compared the secretion of IL-6 and IL-8 from HASM cells infected with RV-16 or exposed to UVi RV-16 for 24 hours. UVi RV stimulated the production of IL-6 from both asthmatic (n = 6, p < 0.05) and non-asthmatic (n = 8, p < 0.05) HASM cells. Of interest, UVi RV-16 induced similar levels of IL-6 when compared to infectious RV-16 (Figure 3A). The secretion of IL-8 protein was significantly increased in only the asthmatic-derived HASM cells (n = 9, p < 0.01) in response to UVi RV, as was found for infection with RV-16 (Figure 3B).

To ensure that IL-6 production in response to UVi RV-16 was not due to another factor in the viral inoculum, in a separate series of experiments using the same viral stocks and the same infection and UV irradiation protocols, IL-6 release was induced by only RV-16 and not UVi RV-16 in alveolar macrophages (data not shown). This suggests that the HASM response to UVi RV-16 is both a cell specific response, and due to ICAM-1 virus interactions.

No difference was observed between the constitutive secretion of eotaxin and that found following exposure to UVi RV-16 in HASM cells derived from asthmatic (UVi RV-16 582.6 ± 247.8, compared to constitutive release 627.2 ± 272.4 pg/ml, p = 0.5, n = 5) and non-asthmatic (UVi RV-16 795.1 ± 281.2 compared to 690.5 ± 231.3 constitutive release pg/ml, p = 0.7, n = 6) subjects.

### IL-6 levels in response to RV are increased in sub-confluent asthmatic HASM cells

**Figure 4:**
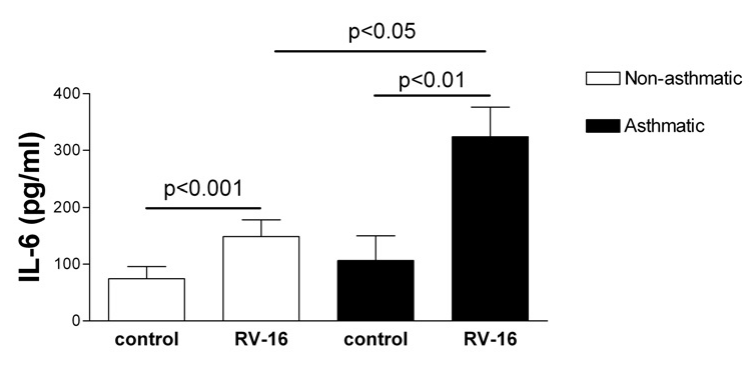
**Cytokine release from infected subconfluent HASM cells**. IL-6 release in response to rhinovirus infection of subconfluent HASM cells, derived from non-asthmatic (open bars) and asthmatic (black bars) donors, measured in the cell free tissue culture supernatant 24 hours post infection. n = 6 both asthmatic and non-asthmatic derived cells.

HASM cells derived from asthmatic subjects have been shown to proliferate faster than cells derived from non-asthmatic subjects [31], however in cells grown to confluence, as used in the above studies, no difference in cell number is found. To exclude the possibility that the difference observed in RV-16 induced IL-6 secretion between cells derived from asthmatic and non-asthmatic subjects was due to differences in cell number, HASM cells were from both groups were seeded at the same density (10^4 ^cells/ml) and infected with RV-16 (MOI 4) 1 day post sub-culture. As was observed in HASM cells grown to confluence, IL-6 secretion was induced 24 hours post RV-16 infection of both sub-confluent asthmatic, and sub-confluent non-asthmatic-derived HASM cells. Furthermore, as in confluent cells, the RV-16-induced IL-6 was significantly greater in HASM cells derived from asthmatic than non-asthmatic derived HASM cells (Figure 4).

### Similar cell surface expression of ICAM-1 on asthmatic and non-asthmatic HASM cells

**Figure 5:**
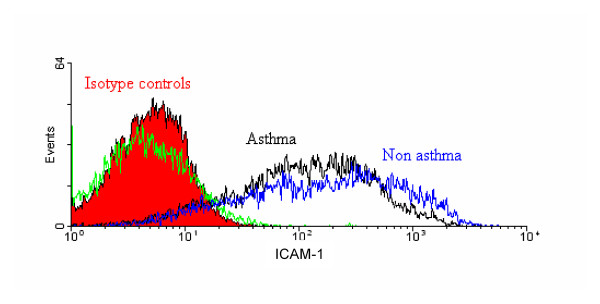
**Similar expression of ICAM-1 on asthmatic and non-asthmatic HASM cells**. A typical histogram of the expression of cell surface ICAM-1 on HASM cells derived from asthmatic (black line) and non-asthmatic (blue line) donors. Isotype controls are represented by the red (solid filled) and green lines for the HASM cells derived from non-asthmatic and asthmatic donors respectively.

Since IL-6 was induced following exposure to UVi RV-16 it is likely that this resulted from interaction between ICAM-1 (RV cellular receptor) and UVi RV-16. To ensure that the increased production of IL-6 observed in the asthmatic derived cells was not due to increased expression of ICAM-1 upon these cells, we measured the cell surface expression of ICAM-1 on asthmatic and non-asthmatic HASM cells. As shown in Figure 5, no significant difference in the constitutive cell surface expression of ICAM-1 was found between asthmatic and non-asthmatic derived HASM cells (asthmatic 230.5 ± 87.57 mean fluorescence ± SEM, n = 4 and non-asthmatic 254.4 ± 46.57 mean fluorescence ± SEM, n = 7 p > 0.05).

### Transcriptional control of IL-6 production in asthmatic, compared to normal HASM cells

We have previously demonstrated that the transcription factor C/EBP-α is absent from asthmatic but not non-asthmatic HASM cells [24]. Having shown that induction of IL-6 is augmented in RV-infected HASM cells from asthmatic donors and in the knowledge that the IL-6 promoter region contains a C/EBP binding site we next investigated transcriptional regulation of IL-6 induction to determine if differences exist between asthmatic and non-asthmatic-derived cells. In HASM cells transfected with the intact human IL-6 promoter construct (NF-κB, C/EBP and AP1 binding sites), significant up regulation of luciferase activity was found in only asthmatic derived HASM cells in response to infectious RV-16 (MOI 0.04). Similarly, at higher MOIs (0.4 and 4) up regulation was observed in only the asthmatic-derived cells (2.5 ± 0.8 and 2.2 ± 0.5 -fold increase respectively, n = 8 for both). In comparison with the positive control (PDGF BB, 10 ng/ml), there was significantly increased luciferase activity of the intact IL-6 promoter construct in both HASM cells derived from asthmatic (p < 0.01, n = 6) and non-asthmatic patients (p < 0.05, n = 7), in comparison to basal luciferase activity (data not shown).

**Figure 6:**
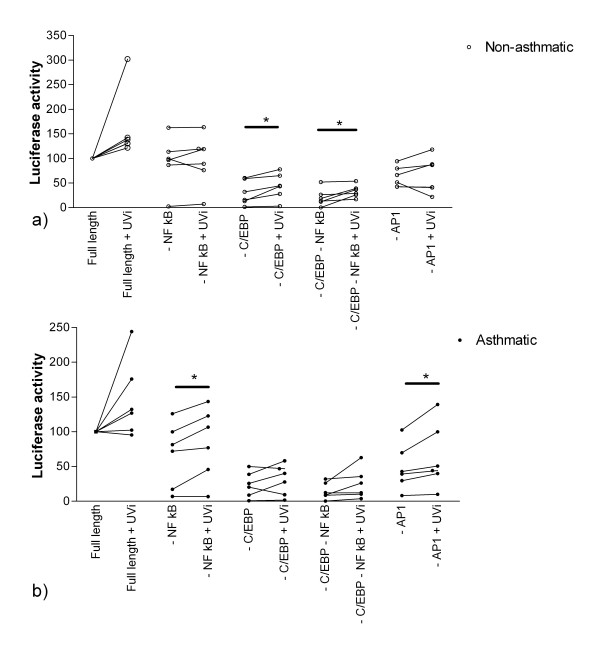
**Transcriptional control of IL-6**. Luciferase activity following exposure to UVi rhinovirus (UVi), of the IL-6 luciferase promoter construct plasmids in a) non-asthmatic derived HASM cells and b) HASM cells derived from asthmatic donors. The IL-6 promoter construct plasmid with no transcription factor deletions is referred to as full length and the various deletions are indicated by a minus sign in front of the transcription factor binding site. All data sets from each donor are normalised to the constitutive expression of the full-length plasmid, which has been assigned the arbitrary value of 100. * indicates p < 0.05 in the luciferase constructs which contained transcription factor binding site deletions in response to UVi RV-16. n = 6 non-asthmatic and n = 5 asthmatic derived HASM cells.

Since UVi RV-16 induced similar IL-6 secretion to live RV-16 without the induction of HASM cell necrosis, and since no up regulation of luciferase activity was observed in HASM cells derived from non-asthmatic subjects in response to infectious RV-16, the transcriptional control of IL-6 production was examined using UVi RV-16. In HASM cells derived from non-asthmatic but not asthmatic donors, significant up-regulation of the luciferase activity of the IL-6 promoters containing C/EBP, and C/EBP and NF-κB deletions was observed. In contrast, luciferase activity was not increased when either NF-κB or AP-1 binding sites were deleted (Figure 6A). In summary these observations indicate that IL-6 production in these cells, in response to exposure to UVi RV-16, is mediated by NF-κB and AP-1 binding sites.

In comparison, significant up-regulation of the IL-6 promoter luciferase activity containing NF-κB/AP-1 deletions and the plasmid containing an AP-1 deletion were observed in HASM cells derived from asthmatic but not non-asthmatic donors (Figure 6B). Therefore, IL-6 production in asthmatic derived HASM cells, in response to exposure to UVi RV, is mediated mainly by transcription factors binding to the C/EBP binding site.

## Discussion

In this study, significant differences were found in the innate responses to RV infection between primary HASM cells derived from asthmatic and non-asthmatic subjects. RV-induced IL-6 release was significantly greater in the asthmatic-derived HASM cells in comparison to cells derived from non-asthmatic subjects. The increased expression of IL-6 in HASM cells obtained from asthma patients was due to differences in the activation pattern of several transcription factors.

The present study investigated pro-inflammatory cytokine production from HASM cells, and represents the first reported experiments to examine RV infection of asthmatic-derived HASM cells. Important differences in the innate responses to RV in asthmatic compared to normal subjects were observed. Hakonarson et al (1988) previously examined RV infection of airway smooth muscle cells [32]. In their studies, they were able to demonstrate that rabbit airway smooth muscle cells exposed to RV exhibited increased constrictor responsiveness to acetylcholine and decreased relaxation to β-adrenoceptor stimulation with isoprenaline. The mechanism by which this occurred was reported to be RV-induced IL-1β, as blocking the IL-1 receptor reversed the changes in contraction and relaxation induced by exposure to RV [22]. However as the evidence for HASM cell production of IL-1β is inconsistent [32], the decision was made in the present study not to measure RV induced IL-1β.

Whilst the purpose of this study was not to determine the kinetics of RV replication within HASM cells, the titer of RV within the cell culture media was measured at 24 hours post-infection. No difference was seen in the level of RV within the cell culture medium derived from HASM cells from asthmatic and non-asthmatic patients. RV has previously been shown to replicate to a higher titre in epithelial cells derived from asthmatic patients [10]. This response appears cell type specific, as it was not observed in HASM cells in the present study.

Greater RV-induced IL-6 secretion occurred in the HASM cells derived from asthmatic patients. This increased release of IL-6 was RV-specific, as the mitogenic stimulus, FBS (in the absence of RV), stimulated similar IL-6 release in the two cell types. Furthermore, the increased IL-6 release was not due to differences in cell cycle status or cell number, as greater RV induced IL-6 release was also observed in sub-confluent HASM cells obtained from asthmatic subjects. The increased IL-6 was also not due to intrinsic differences in ICAM-1 expression between the two cell types. The greater production of IL-6 which occurred in HASM cells derived from asthmatic subjects in comparison to non-asthmatic subjects is most like due to differences in the transcriptional control of IL-6. In common with IL-6 production in response to 5% FBS, UVi RV-induced IL-6 release in both cell types. As occurred with 5% FBS, there was no difference in the amount of IL-6 produced between the two diagnostic groups, further suggesting that whilst IL-6 production is induced by virus receptor interactions, the HASM cells derived from asthmatic patients respond differently to infectious RV resulting in a greater release of IL-6. Whilst it is likely that the majority of IL-6 induction is due to activation of ICAM-1, up-regulation of the luciferase activity of the intact IL-6 promoter construct occurred in only asthmatic derived HASM cells. In parallel experiments, carried out at the same time, stimulation with PDGF BB resulted in up-regulation of the luciferase activity of the full-length IL-6 promoter construct plasmid in both cell types. Therefore, it can be assumed that the increased RV-induced IL-6 in the asthmatic derived HASM cells is as a result of a greater induction of IL-6-specific transcription factors. The increased IL-6 secretion in response to RV infection from asthmatic-derived HASM cell was also observed in sub-confluent cells. This finding confirmed the fact that the increased IL-6 protein production in asthmatic HASM cells is not due to differences in cell number at the time of infection, and moreover, is not dependent on differences in cell cycle status.

IL-6 transcriptional regulation involves at least 4 different transcription factor binding sites [33]: cAMP response element binding protein (CREB), CCAAT/enhancer-binding protein (C/EBP) binding site, activator protein (AP)-1, and nuclear factor (NF)-κB. The transcriptional control of IL-6 production in the asthmatic and non-asthmatic HASM cells following exposure to UVi RV was found to differ. IL-6 protein production in the non-asthmatic-derived HASM cells was primarily mediated by the transcription factor binding sites, NF-κB and AP-1, whilst in the asthmatic derived HASM cells IL-6 production was primarily driven by transcription factors binding to the C/EBP binding site. It has recently been shown that asthmatic HASM cells have a dysfunctional expression of C/EPB α [24], which may account for the greater rate of proliferation observed in asthmatic HASM cells [31]. In general, C/EBP-α is considered to be a negative regulator of protein transcription, therefore the absence in the asthmatic HASM cells may disrupt the balance between excitatory and inhibitory C/EBPs, such that there are more pro-transcriptional members. Thus the role of the C/EBP proteins in these cells upon specific stimulation may be skewed towards pro-transcription. In a report by Ammit and coworkers (2002), HASM cells from non-asthmatic donors in response to stimulation with TNF-α produce IL-6 through transcription factors binding to the NF-κB binding site, whilst AP-1 was shown not to be involved [34]. In a different cell type, IL-6 production in response to RV infection of the epithelial cell line A549 is also mediated via NF-κB [35]. Our findings, in the non-asthmatic HASM cells, support the role of NF-κB in RV-induced IL-6 release.

Further differences between asthmatic and non-asthmatic HASM cells were found upon infection with RV. Whilst 5% FBS stimulated IL-8 secretion, with this being increased by concurrent RV infection in both asthmatic and non-asthmatic cells, under basal conditions, both infectious RV and UVi RV induced release of IL-8 in HASM cells derived from asthmatic donors only.

Several reports have shown that the interaction of RV capsid protein and ICAM-1 can induce a cell-mediated inflammatory response, independent of RV replication. This response has been found in a variety of cell types such as epithelial cells [36], T cells [37], neutrophils [38], monocytes [39] and importantly in the context of this study airway smooth muscle cells [40]. In response to UVi RV, increased IL-8 protein production was observed in the HASM cells derived only from asthmatic donors. This is not surprising since RV-induced IL-8 protein production occurred only in asthmatic HASM cells. Since ICAM-1 is stimulated by both infectious and UVi RV [40] it is likely that IL-8 production in the HASM cells is mediated by RV ICAM-1 interactions. Eotaxin was not found to be induced by either infectious RV or UVi RV, in contrast to epithelial cells, which produce eotaxin in response to RV infection [41]. However, eotaxin is also not induced by RV infection of lung fibroblasts (P. Bardin personal communication).

Viral replication is dependant upon evading detection by the host's immune system. In this study we have shown that both IL-6 and IL-8 are induced by RV infection, and furthermore, the production of IL-6 is increased in HASM cells derived from asthmatic patients. This indicates the important role of the HASM cell as a modulator of the local immune response. Whilst IL-6 has not been shown to be directly antiviral, IL-6 can stimulate endothelial cells to release IL-8, and therefore can contribute to the neutrophilic infiltration observed in viral respiratory tract infections.

The association between asthma exacerbations and viral infection has been known for at least the last 30 years [42-44], and with the recent advances in molecular biology there is now an overwhelming body of evidence in support of virus infections being the major trigger for asthma exacerbations. The exact mechanisms behind viral infection and asthma exacerbations are not understood, although the recent report of increased RV replication and epithelial cell death following RV infection of bronchial epithelial cells from asthmatic subjects [10] suggests increased likelihood of RV infection of ASM in asthmatic subjects. The augmented pro-inflammatory cytokine release we have observed from asthmatic HASM cells is therefore likely an important contribution to increased airway inflammation associated with asthma exacerbations.

Independently of productive infection, both IL-6 and IL-8 protein release were observed in the HASM cells derived from asthmatic donors. These important mediators are not only found to be elevated in the asthmatic airway [45,46], but are also induced upon in vivo RV infections [17,47], suggesting they contribute to the process of exacerbation, through recruitment and activation of neutrophils, as well as perhaps other cells such as eosinophils and mast cells.

## Conclusion

This study is the first to demonstrate differential regulation of virus induced pro-inflammatory cytokines in HASM cells from asthmatic and normal volunteers. The observations reported herein have important implications for the development of new therapies for virus induced asthma exacerbations.

## Competing interests

The author(s) declare that they have no competing interests.

## Authors' contributions

BGGO: carried out the bench work, and drafted the manuscript

SLJ: conceived of the study, and participated in its design and coordination and helped to draft the manuscript. MB: carried out bronchoscopic biopsies and subject recruitment and helped to draft the manuscript. JKB, NJCK, SL participated in the study design and coordination and helped to draft the manuscript. MR: helped with initial luciferase assays, participated in the study design and coordination and helped to draft the manuscript. JLB conceived of the study, and participated in its design and coordination and helped to draft the manuscript. All authors read and approved the final manuscript.
